# Possible Roles of Carbohydrate Management and Cytokinin in the Process of Defoliation–Regrowth Cycles in Rice

**DOI:** 10.3390/ijms25105070

**Published:** 2024-05-07

**Authors:** Yuki Sakashita, Hikaru Kurashima, Mika Fukuda, Haru Hirano, Sagar Lamsal, Naoki Katayama, Takeshi Fukao

**Affiliations:** Department of Bioscience and Biotechnology, Fukui Prefectural University, Fukui 910-1195, Japan

**Keywords:** cytokinin, *Oryza sativa*, photosynthesis, regrowth vigor, sucrose synthase

## Abstract

Defoliation is an inevitable abiotic stress for forage and turf grasses because harvesting, grazing, and mowing are general processes for their production and management. Vegetative regrowth occurs upon defoliation, a crucial trait determining the productivity and persistence of these grasses. However, the information about the molecular regulation of this trait is limited because it is still challenging to perform molecular analyses in forage and turf grasses. Here, we used rice as a model to investigate vegetative regrowth upon defoliation at physiological and molecular levels. This study analyzed stubble and regrown leaves following periodic defoliation using two rice varieties with contrasting regrowth vigor. Vigorous regrowth was associated with maintained chlorophyll content and photosystem II performance; a restricted and promoted mRNA accumulation of sucrose synthase (SUS) I and III subfamilies, respectively; and reduced enzymatic activity of SUS. These results suggest that critical factors affecting vegetative regrowth upon defoliation are de novo carbohydrate synthesis by newly emerged leaves and proper carbohydrate management in leaves and stubble. Physiological and genetic analyses have demonstrated that the reduced sensitivity to and inhibited biosynthesis of cytokinin enhance regrowth vigor. Proper regulation of these metabolic and hormonal pathways identified in this study can lead to the development of new grass varieties with enhanced regrowth vigor following defoliation.

## 1. Introduction

Defoliation is the premature removal of leaves by cutting or grazing, an inevitable abiotic stress for forage and turf grasses. Upon defoliation, new leaves emerge from the remaining stubble, defined as vegetative regrowth, a critical trait influencing the productivity and persistence of these grasses. Many physiological studies have been performed to elucidate the regulation of this commercially important trait. Defoliation eliminates most photosynthetic tissues, substantially reducing or even shutting down carbohydrate production by photosynthesis. In this situation, the emergence of new leaves depends on carbohydrate reserves in bermudagrass (Cynodon dactylon), perennial ryegrass (Lolium perenne), and tall fescue (Festuca arundinacea) [1,2,3]. The amount of stubble left after defoliation reflects the level of carbohydrate reserves, a critical factor affecting regrowth vigor. In orchardgrass (Dactylis glomerata), perennial ryegrass, and timothy (Phleum pratense), low cutting height (i.e., intensive defoliation) resulted in significant decreases in vegetative regrowth, relative to high cutting height (i.e., moderate defoliation) [4,5].

In addition to carbohydrates stored before defoliation, those newly synthesized in emerged leaves also play a critical role in regrowth vigor. Radioactive tracer experiments in perennial ryegrass revealed that leaf elongation upon defoliation relied on carbohydrate reserves only in the initial regrowth stage (0–3 days after defoliation), and carbohydrates newly synthesized in emerged leaves were significant for vegetative regrowth 3 days or more after defoliation [6]. The dependence of vegetative regrowth on carbohydrates stored in the remaining tissues may be linked to the amount of photosynthetic tissues left after defoliation. When less than 65% of the leaf area was removed from perennial ryegrass, defoliated plants did not need the recruitment of carbohydrate storage compounds to growing leaves [7]. Thus, when sufficient photosynthetic tissues remain in defoliated plants, carbohydrate reserves are not utilized to support vegetative regrowth.

Molecular studies have uncovered various regulatory mechanisms underlying plant growth and development. However, molecular approaches have been rarely utilized for vegetative regrowth because plant species used to investigate vegetative regrowth are not suitable for molecular studies. Generally, forage and turf grasses have self-incompatibility, polyploidy, limited mutant collections, and/or inadequate molecular information/tools, which are major barriers to molecular analyses [8,9,10].

In the present study, we used rice (*Oryza sativa* L.) as a model to investigate vegetative regrowth after defoliation. Rice has been used as a model, enhancing our understanding of the biology in grasses [11,12]. Unlike other annual cereals, rice has a relatively strong regrowth vigor, allowing farmers to harvest a second crop from a single plant [13,14]. Rice is suitable for molecular studies because this grass has self-compatibility, a small genome with high-quality sequences and annotations, a wealth of mutant collections, and various molecular information/tools [15,16,17]. These characteristics make it possible to dissect regulatory mechanisms of vegetative regrowth upon defoliation at the molecular level, unfeasible for forage and turf grasses. We hypothesize that regrowth vigor is regulated by the proper management of carbohydrate breakdown and photosynthesis. We also evaluated the involvement of cytokinin in vegetative regrowth. These hypotheses were tested by a combination of physiological and molecular approaches, shedding new insights into the underlying mechanism of the critical but understudied trait in grasses.

## 2. Results

### 2.1. Vegetative Regrowth after Periodic Defoliation in Rice

**Figure 1:**
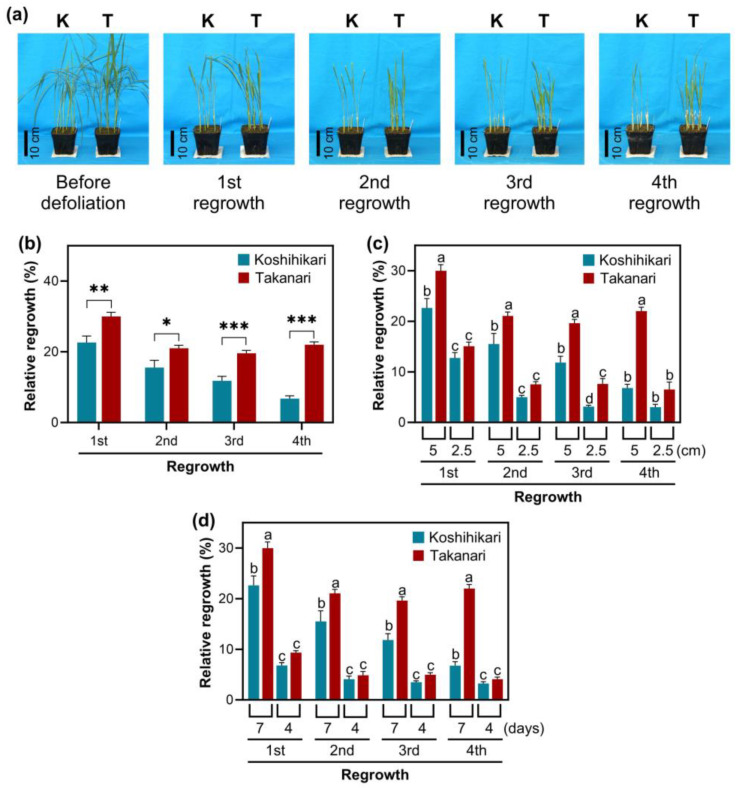
Regrowth vigor after defoliation in rice. (**a**) Photos of rice plants regrown after defoliation. Plants were exposed to weekly clipping at 5 cm cutting height and regrown for 7 days. K, Koshihikari; T, Takanari. (**b**) Relative regrowth of Koshihikari and Takanari after defoliation cycles. Relative regrowth was calculated by comparison with the dry weight of leaves clipped at the first defoliation in each variety. The data represent means ± SE from four replicates. Asterisks indicate significant differences between the two varieties (*t*-test; * *p* < 0.05, ** *p* < 0.01, *** *p* < 0.001). (**c**) Relative regrowth of Koshihikari and Takanari after defoliation at 5 or 2.5 cm cutting height. Plants were exposed to defoliation cycles every 7 days at distinct cutting heights. (**d**) Relative regrowth of Koshihikari and Takanari after defoliation at a 7- or 4-day interval. Plants were exposed to defoliation cycles at 5 cm cutting height at distinct regrowth intervals. In (**c**,**d**), Data represent means ± SE from four replicates. Bars not sharing the same letter significantly differ in each defoliation cycle (ANOVA followed by Tukey HSD test; *p* < 0.05).

This study investigated the degree of vegetative regrowth after periodic defoliation in two rice varieties, Koshihikari and Takanari, in the vegetative stage (Figure 1a). Koshihikari is a premium *japonica* cultivar with superior eating quality and sticky texture [18]. Koshihikari is not recognized as a high-yielding variety. Takanari is an *indica* cultivar that exhibited the highest yield record in Japan [19]. Takanari is not categorized into high-eating-quality varieties. Koshihikari and Takanari were used as parental lines to establish reciprocal Chromosome Segment Substitution Lines (CSSLs) [19], which facilitate the identification of chromosomal segments associated with regrowth vigor after defoliation. In every cutting cycle, the dry weight and area of harvested leaves were more significant in Takanari than in Koshihikari (Figure 1b and Figure S1a), indicating that Takanari exhibited a greater regrowth vigor. The effect of cutting height and interval on vegetative regrowth was assessed in Takanari and Koshihikari. Plants cut to 2.5 cm displayed a lower dry weight and area of harvested leaves than those cut to 5 cm in both Takanari and Koshihikari in each cutting cycle (Figure 1c and Figure S1b), suggesting that the size of stubble positively correlates with the degree of vegetative regrowth upon defoliation. Regarding cutting frequency, plants cut in a 4-day interval showed a lower dry weight of harvested leaves than those cut in a 7-day interval in both varieties in all cutting cycles (Figure 1d), although this trend was observed only in the fourth regrowth cycle when the area of harvested leaves was analyzed (Figure S1c). These data indicate that the length of a cutting interval is also linked with the degree of vegetative regrowth.

### 2.2. Carbohydrate Management in Rice Exposed to Periodic Defoliation

**Figure 2:**
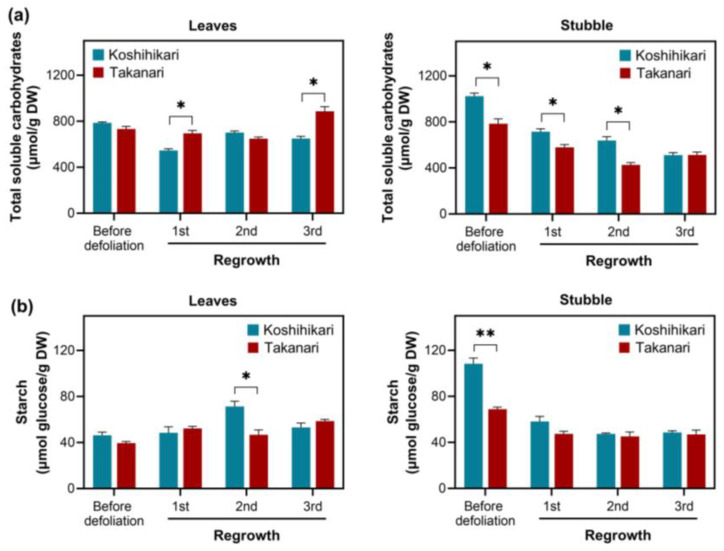
The amount of carbohydrate reserves in leaves and stubble of rice plants exposed to periodic defoliation (5 cm cutting height, 7 d interval). Total soluble carbohydrates (**a**) and starch (**b**) were quantified in leaves and stubble in each defoliation cycle. Data represent means ± SE from three replicates. Asterisks indicate significant differences between the two varieties (*t*-test; * *p* < 0.05, ** *p* < 0.01).

Clipping at 2.5 and 5 cm cutting heights eliminates all leaf blades in rice, thus resulting in the loss of photosynthetic tissues. In this situation, defoliated rice relies on carbohydrate reserves in the remaining stubble as energy sources for vegetative regrowth. Short cutting height significantly reduced regrowth vigor upon defoliation (Figure 1c and Figure S1b), supporting the idea that carbohydrate reserves in the stubble are vital for vegetative regrowth. To monitor the content of carbohydrate reserves in leaves and stubble over the cutting–regrowth cycles, total soluble carbohydrates and starch were quantified in these tissues of plants exposed to periodic defoliation (5 cm cutting height and 7 d interval) (Figure 2). In leaves and stubble, the levels of total soluble carbohydrates were considerably more significant than those of starch. The total soluble carbohydrates in leaves were greater in Takanari than in Koshihikari in the 1st and 3rd regrowth cycles (Figure 2a). In contrast to leaves, the content of total soluble carbohydrates in stubble was larger in Koshihikari than in Takanari in most regrowth cycles. The starch content in leaves was more significant in Koshihikari than in Takanari only during the 2nd regrowth cycle (Figure 2b). The same trend was observed in stubble before defoliation. It appears that leaves and stubble have distinct roles in carbohydrate accumulation. In leaves, the total soluble carbohydrates and starch levels remained stable over the regrowth cycles. However, in stubble, the contents of both carbohydrate reserves declined by defoliation.

### 2.3. Gene Expression and Activities of Enzymes Associated with Carbohydrate Management in Rice Exposed to Periodic Defoliation

Sucrose is the end product of photosynthesis and the primary sugar reserved and transported in vegetative tissues of rice [20,21]. Sucrose synthase is a crucial enzyme for sucrose degradation and is recognized as a biochemical marker for sink strength [22]. The rice genome encodes seven sucrose synthase genes (*SUSs*). In this study, we monitored the mRNA accumulation of all sucrose synthase genes in leaves and stubble over periodic defoliation.

**Figure 3:**
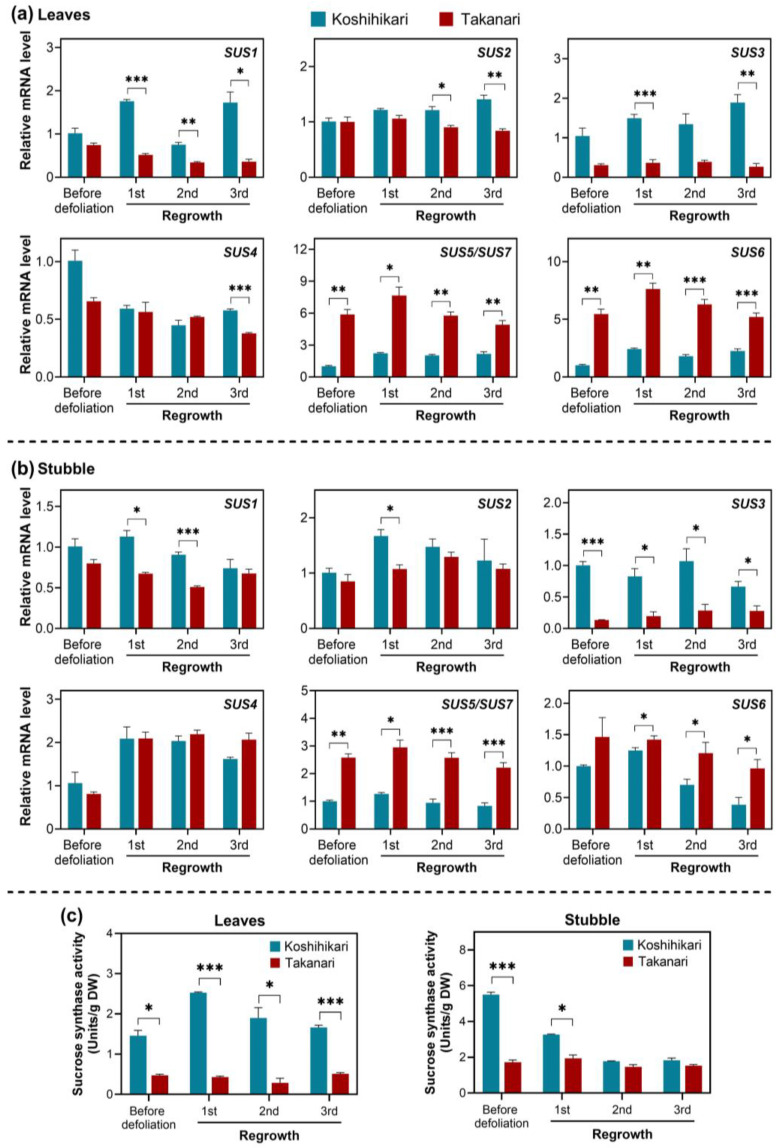
Gene expression and enzymatic activity of sucrose synthase in leaves and stubble of rice plants exposed to periodic defoliation (5 cm cutting height, 7 d interval). Relative mRNA levels of genes encoding sucrose synthase were assessed by quantitative RT-PCR in leaves (**a**) and stubble (**b**) in each defoliation cycle. Relative mRNA levels were calculated by comparison with the level in Koshihikari on day 0 (set at 1.0). (**c**) The enzymatic activity of sucrose synthase was assayed in leaves and stubble in each defoliation cycle. Data represent means ± SE from three replicates. Asterisks indicate significant differences between the two varieties (*t*-test; * *p* < 0.05, ** *p* < 0.01, *** *p* < 0.001).

Because *SUS5* has a 99.6% similarity with *SUS7* in terms of nucleotide sequences, gene-specific primers distinguishing these *SUS*s could not be designed. Therefore, the mRNA accumulation of these genes was analyzed using a single primer pair recognizing both transcripts. In leaves, *SUS1*, *SUS2*, *SUS3*, and *SUS4* mRNAs were highly accumulated in Koshihikari, relative to Takanari, in some regrowth cycles (Figure 3a). The other *SUS* genes, *SUS5* and *SUS6*, were considerably expressed in Takanari, compared to Koshihikari, over periodic defoliation. Similar to leaves, *SUS1*, *SUS2*, and *SUS3* mRNAs in stubble are more highly expressed in Koshihikari than in Takanari, although no differences in *SUS4* mRNA levels between the two varieties were detected (Figure 3b). The transcript levels of *SUS5* and *SUS6* in stubble were greater in Takanari than in Koshihikari, consistent with the results in leaves. 

The enzymatic activity of sucrose synthase was determined in the direction of sucrose degradation in leaves and stubble of rice plants exposed to periodic defoliation (Figure 3c). In leaves, the sucrose synthase activity was considerably higher in Koshihikari than in Takanari in every regrowth cycle. The same trend was observed in stubble only before defoliation and in the first regrowth cycle. 

**Figure 4:**
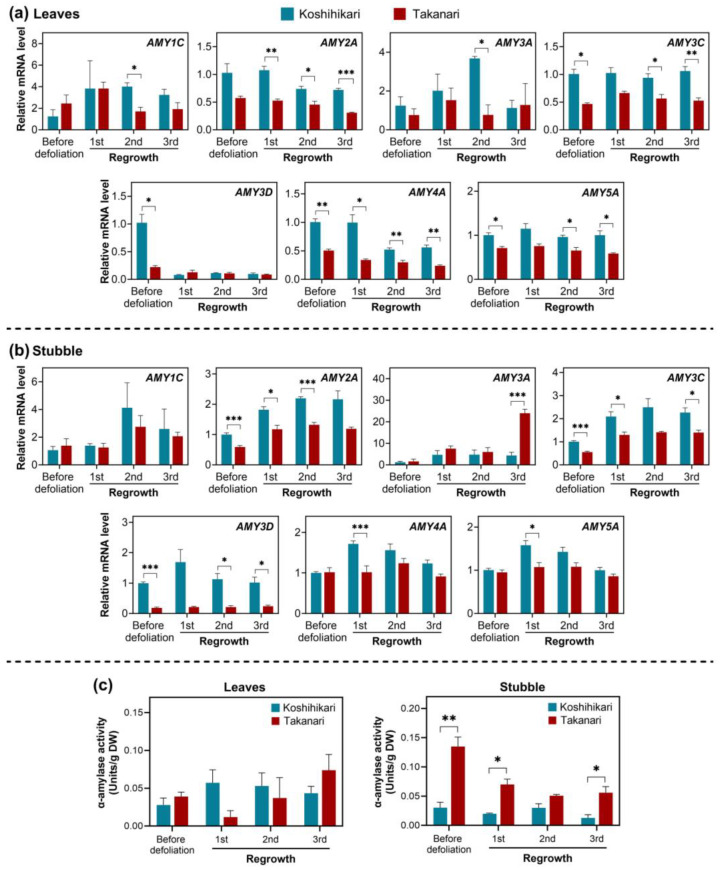
Gene expression and enzymatic activity of α-amylase in leaves and stubble of rice plants exposed to periodic defoliation (5 cm cutting height, 7 d interval). Relative mRNA levels of genes encoding α-amylase were assessed by quantitative RT-PCR in leaves (**a**) and stubble (**b**) in each defoliation cycle. Relative mRNA levels were calculated by comparison with the level in Koshihikari on day 0 (set at 1.0). (**c**) Enzymatic activity of α-amylase was assayed in leaves and stubble in each defoliation cycle. Data represent means ± SE from three replicates. Asterisks indicate significant differences between the two varieties (*t*-test; * *p* < 0.05, ** *p* < 0.01, *** *p* < 0.001).

Although rice accumulates sucrose rather than starch in leaves, starch is still a critical carbohydrate storage [23]. Among the enzymes involved in starch breakdown, α-amylases are the major ones, and the rice genome contains 11 α-amylase genes (*AMYs*) [24,25]. In the present study, seven *AMY* genes expressed in leaves and stubble were analyzed for their expression levels in rice plants exposed to periodic defoliation. All *AMY* genes surveyed in leaves were highly expressed in Koshihikari, relative to Takanari, before defoliation or during at least one defoliation cycle (Figure 4a). In stubble, this trend was observed in *AMY2A*, *AMY3C*, *AMY3D*, *AMY4A*, and *AMY5A* (Figure 4b). The mRNA abundance of *AMY3A* was higher in Takanari than in Koshihikari in the third regrowth cycle.

Regarding the enzymatic activity of α-amylase, no differences between the two varieties were detected in leaves (Figure 4c). In stubble, however, Takanari displayed a higher α-amylase activity than Koshihikari at most time points. 

### 2.4. Effect of Photosynthesis on Vegetative Regrowth upon Defoliation in Rice

**Figure 5:**
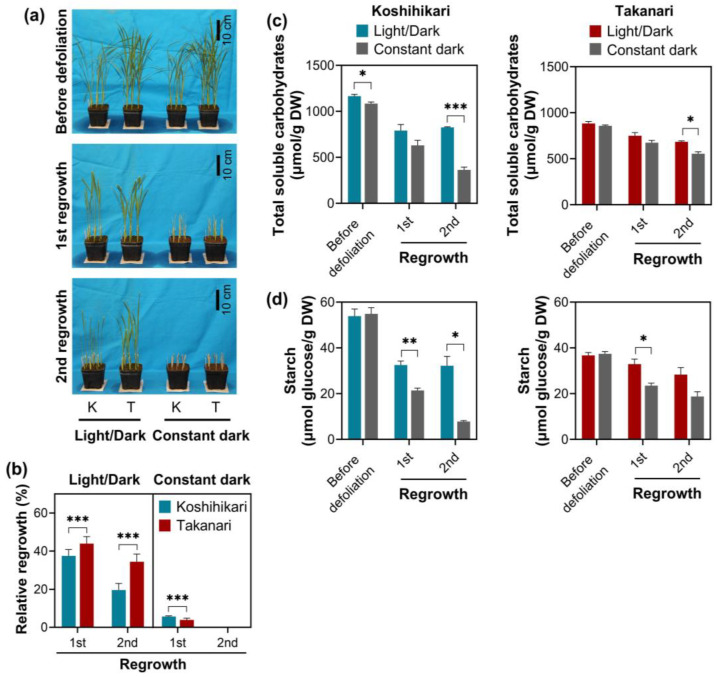
Vegetative regrowth after periodic defoliation (5 cm cutting height, 7 d interval) in rice under light-dark cycles and constant darkness. (**a**) Photos of rice plants regrown after weekly defoliation under light-dark cycles and constant darkness. K, Koshihikari; T, Takanari. (**b**) Relative regrowth of rice plants exposed to weekly defoliation under light-dark cycles and constant darkness. Relative regrowth was calculated by comparison with the dry weight of leaves clipped at the first defoliation in each variety. Data represent means ± SE from four replicates. Asterisks indicate significant differences between the two varieties (*t*-test; *** *p* < 0.001). The amount of total soluble carbohydrates (**c**) and starch (**d**) in stubble of rice plants exposed to defoliation under light-dark cycles and constant darkness. This experiment did not analyze leaves because sufficient leaves did not emerge from the stubble under constant darkness. Data represent means ± SE from three replicates. Asterisks indicate significant differences between the two light conditions (*t*-test; * *p* < 0.05, ** *p* < 0.01, *** *p* < 0.001).

To evaluate the effect of photosynthesis on vegetative regrowth, plants grown under regular growth conditions were defoliated and subsequently recovered under 12 h light and 12 h dark cycles (regular growth conditions) or constant darkness (Figure 5). Constant darkness drastically repressed the vegetative regrowth of both varieties after the first defoliation as compared to light–dark cycles (Figure 5a,b). Under light–dark cycles, Takanari exhibited more vigorous regrowth than Koshihikari. Conversely, under constant darkness, Koshihikari showed active regrowth relative to Takanari. Upon the second defoliation, leaves did not emerge from the stubble in either variety under constant darkness. These observations support the notion that photosynthesis by regrown leaves is vital for vegetative regrowth upon defoliation.

The levels of carbohydrate reserves were monitored in the stubble of rice plants exposed to periodic defoliation under light–dark cycles and constant darkness. In this experiment, leaves were not subjected to carbohydrate assays because sufficient leaves did not emerge under constant darkness. Constant darkness promoted the consumption of total soluble carbohydrates and starch in stubble of both accessions (Figure 5c,d). Interestingly, reductions in total soluble carbohydrates and starch under constant darkness were more evident in Koshihikari than in Takanari. These data are consistent with the observation that Koshihikari displayed more active regrowth than Takanari under constant darkness.

**Figure 6:**
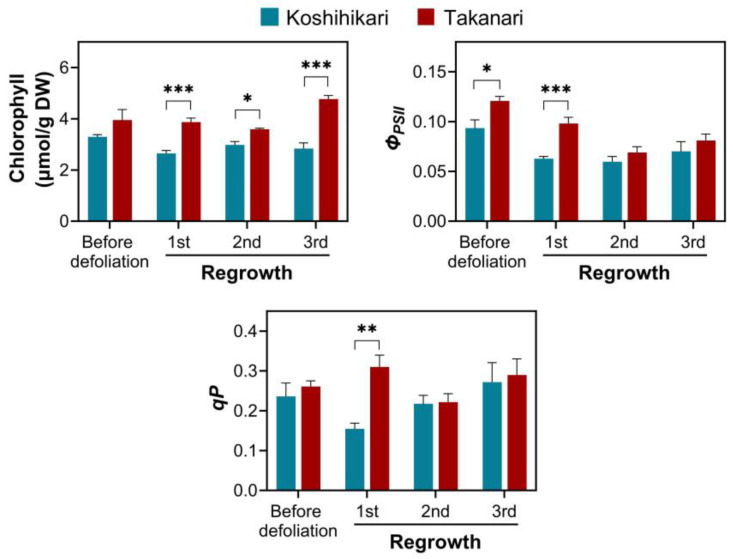
The chlorophyll content and chlorophyll fluorescence in leaves of rice plants exposed to periodic defoliation (5 cm cutting height, 7 d interval). The chlorophyll content was quantified using leaf tissues clipped weekly. The effective quantum yield of photosystem II (*Φ_PSII_*) and photochemical quenching (*qP*) were measured in the uppermost leaves of plants recovered for 7 days. Data represent means ± SE from three replicates for the chlorophyll content and six replicates for *Φ_PSII_* and *qP*. Asterisks indicate significant differences between the two varieties (*t*-test; * *p* < 0.05, ** *p* < 0.01, *** *p* < 0.001).

To further examine the significance of photosynthesis in vegetative regrowth, we investigated the photosynthetic capability of regrown leaves in rice exposed to periodic defoliation (Figure 6). The amount of chlorophyll in leaves was not different between Koshihikari and Takanari before defoliation, but it was significantly higher in Takanari than in Koshihikari in every regrowth cycle. Regarding photosystem II performance, the effective quantum yield of photosystem II (*Φ_PSII_*) in leaves was more significant in Takanari than in Koshihikari before defoliation, and this trend was continuously observed in the first regrowth cycle. Photochemical quenching (*qP*) was also higher in Takanari than in Koshihikari in the first regrowth cycle. These data suggest that photosynthetic capability is associated with regrowth vigor, especially in the early stage of periodic defoliation. 

### 2.5. Effect of Cytokinin on Vegetative Regrowth after Defoliation in Rice

**Figure 7:**
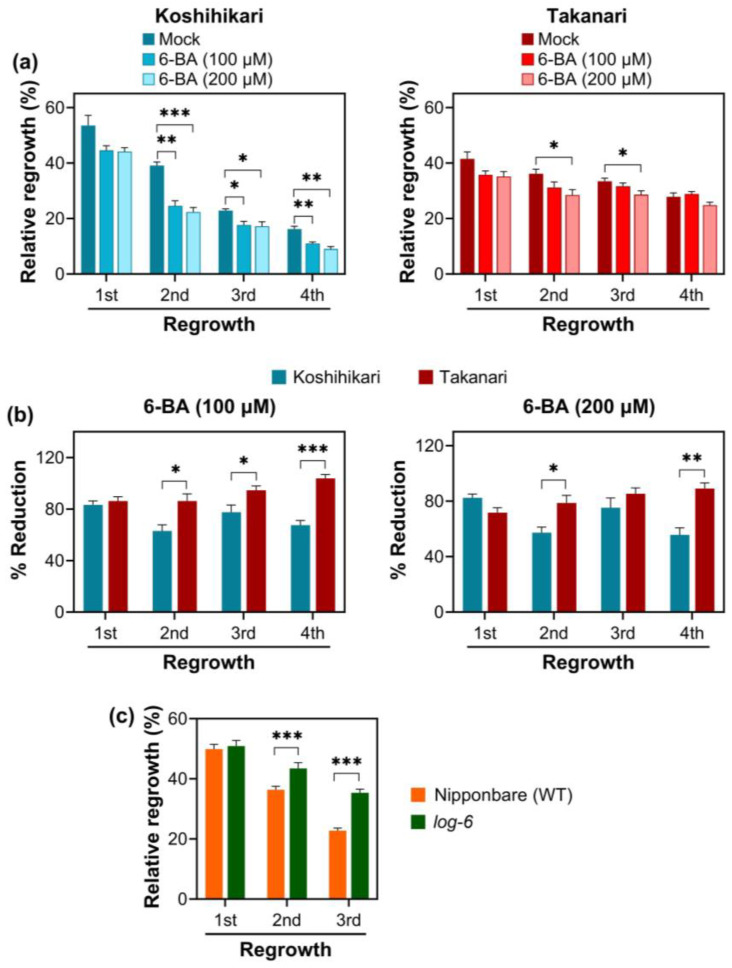
Effect of cytokinin on vegetative regrowth after periodic defoliation. (**a**) Fourteen-day-old plants were exposed to weekly clipping at 5 cm cutting height and regrown for 7 days. The defoliation-regrowth cycles were repeated four times. Upon the initial defoliation, mock, 100, or 200 µM 6-benzyl adenine (6-BA; a synthetic cytokinin) was sprayed to plants daily until the end of the 4th regrowth. Data represent means ± SE from four replicates. Asterisks indicate significant differences between mock and 6-BA-treated samples (*t*-test; * *p* < 0.05, ** *p* < 0.01, *** *p* < 0.001). (**b**) Percent reduction in relative regrowth by 6-BA treatment was calculated by dividing the relative regrowth of 6-BA (100 or 200 µM) treated plants by the relative regrowth of mock-treated plants in each defoliation cycle. Data represent means ± SE from four replicates. Asterisks indicate significant differences between the two varieties (*t*-test; * *p* < 0.05, ** *p* < 0.01, *** *p* < 0.001). (**c**) Fourteen-day-old plants were exposed to weekly clipping at 5 cm cutting height and regrown for 7 days. The defoliation-regrowth cycles were repeated three times. *log-6* is a loss-of-function mutant of *LONELY GUY*, a cytokinin-activating enzyme, in the Nipponbare background. Data represent means ± SE from four replicates. Asterisks indicate significant differences between the two lines (*t*-test; *** *p* < 0.001).

Most traits involved in plant growth and development are regulated by phytohormones. Vegetative regrowth is also a growth-related trait that a phytohormone would promote or suppress. Of various hormones, cytokinin regulates shoot apical meristem (SAM) activity, energy metabolism, and transport [26,27], a strong candidate for controlling vegetative regrowth upon defoliation. To evaluate the influence of cytokinin in vegetative regrowth, a synthetic cytokinin, 6-benzyl adenine (6-BA), was sprayed on rice plants exposed to periodic defoliation. The exogenous application of 6-BA suppressed regrowth vigor, especially in Koshihikari (Figure 7a). Significant reductions in vegetative regrowth were observed in Takanari only when 200 µM 6-BA was sprayed, but 100 µM 6-BA was sufficient for hampered regrowth in Koshihikari. To assess the sensitivity of the two varieties to 6-BA more accurately, percent reductions in vegetative regrowth by 6-BA were compared between the two varieties (Figure 7b). At both 100 and 200 µM 6-BA, vegetative regrowth in Koshihikari was more significantly suppressed than Takanari, indicating that Koshihikari is more sensitive to cytokinin than Takanari.

Exogenous cytokinin application reduced vegetative regrowth upon defoliation, indicating that cytokinin is a negative regulator for this trait. To further support this notion, a cytokinin biosynthesis mutant, *lonely guy-6* (*log-6*), was exposed to periodic defoliation (Figure 7c). LOG is a phosphoribohydrolase-activating enzyme that converts a cytokinin nucleotide to an active free-base form of cytokinins in the final step of cytokinin synthesis. The rice *log-6* mutant lacks the ability to synthesize bioactive cytokinins [28]. The present study showed that weekly defoliation gradually reduced vegetative regrowth in wild-type Nipponbare. However, *log-6* maintained regrowth vigor during periodic defoliation cycles. This result is in accordance with the observation that exogenous cytokinin application reduced vegetative regrowth. Altogether, it is concluded that cytokinin can serve as a negative regulator for vegetative regrowth upon defoliation. 

## 3. Discussion

Vegetative regrowth upon defoliation is a major agronomical trait that substantially influences the yield and persistence of forage and turf grasses. However, molecular aspects of vegetative regrowth remain unclear, because self-incompatibility, polyploidy, and limited molecular information and tools make it difficult to investigate these grasses at the molecular level [8,9,10]. In this study, we applied rice as a model to study vegetative regrowth upon defoliation because this grass species has diverse regrowth vigor among varieties, self-compatibility, small genome size, and a wealth of molecular information and tools [15,16,17]. 

A short cutting height (2.5 cm) significantly reduced vegetative regrowth upon defoliation in Takanari and Koshihikari relative to a long cutting height (5 cm) (Figure 1c and Figure S1b). These results reflect that the abundance of energy reserves in stubble, including soluble carbohydrates and starch, affects regrowth vigor as observed in other forage and turf grasses [1,2,3]. Total soluble carbohydrate and starch levels in stubble reduced over the defoliation–regrowth cycles, whereas these carbohydrate reserves remained stable in leaves, which can generate carbohydrates by photosynthesis (Figure 2). It appears that the abundance of total soluble carbohydrates and starch in stubble dropped more significantly in Koshihikari than in Takanari, which is inconsistent with the degree of vegetative regrowth in these varieties (Figure 1b, Figure 2 and Figure S1a). Reductions in carbohydrate reserves do not necessarily indicate the degree of carbohydrate consumption, because this analysis merely determines the steady-state levels of carbohydrate reserves. Vigorous vegetative regrowth in Takanari may be attributable to high carbohydrate consumption, which is allowed by high carbohydrate production via photosynthesis, relative to Koshihikari. A more abundant chlorophyll content and higher photosystem II performance in Takanari than in Koshihikari (Figure 6) support this notion. The constant-darkness experiments verified the significance of photosynthesis in vegetative regrowth (Figure 5). These results emphasize that photosynthesis by newly emerged leaves plays a pivotal role in vegetative regrowth. Overall, it is likely that vigorous vegetative regrowth in Takanari results from active photosynthesis by newly emerged leaves, which may allow high carbohydrate consumption. 

Unlike forage and turf grasses, high-quality, well-annotated genome sequences are available in rice. Taking advantage of the genomic information in the model species, we surveyed the complete set of sucrose synthase genes regarding mRNA accumulation (Figure 3). In plants, sucrose synthase genes are classified into three subfamilies based on phylogenetic analysis [22]. In rice, *SUS1*, *SUS2*, and *SUS3* belong to the SUS I subfamily. *SUS4* is the sole member of the SUS II subfamily. *SUS5*, *SUS6*, and *SUS7* are members of the SUS III subfamily. In the present study, all SUS I gene transcripts (*SUS1-3*) tended to be highly accumulated in leaves and stubble of Koshihikari, relative to Takanari. On the other hand, the SUS III gene sub-family (*SUS5-7*) exhibited higher expression in Takanari than in Koshihikari at most time points. These data have demonstrated that the reduced SUS I mRNA and increased SUS III mRNA levels positively correlate with vigorous regrowth upon defoliation. The antithetical regulation of SUS I and SUS III gene expression may be crucial for fine-tuning sucrose synthase activity and carbohydrate management in leaves and stubble of defoliated plants. 

Surveying the expression patterns of all sucrose synthase genes in Arabidopsis revealed that the SUS III subfamily (*AtSUS5* and *AtSUS6*) was expressed only in specific tissues, whereas the SUS I and SUS II subfamilies exhibited broader expressions [22]. The intron/exon structures of SUS III genes were also unique compared with those of SUS I and SUS II. Regarding protein accumulation, the Arabidopsis SUS III subfamily was specifically present in the phloem [29]. These data imply that the rice SUS III subfamily may also be expressed in the phloem, contributing to increased sink strength [21]. Indeed, a higher accumulation of SUS III subfamily mRNAs in Takanari than in Koshihikari over the defoliation–regrowth cycles is associated with more active regrowth vigor following defoliation (Figure 1 and Figure 3).

We also investigated the mRNA accumulation of all α-amylase genes expressed in vegetative tissues (Figure 4). The rice genome encodes 11 α-amylase genes (*AMY1A*, *AMY1B*, *AMY1C*, *AMY2A*, *AMY3A*, *AMY3B*, *AMY3C*, *AMY3D*, *AMY3E*, *AMY4A*, and *AMY5A*), which are categorized into five subfamilies (*AMY1*-*5*) [24,25]. The tissue and organ specificity of *AMY* gene expression is not linked with subfamilies [24]. The present study investigated the mRNA accumulation of all *AMY* genes expressed in leaves and stubble (Figure 4). The transcript accumulation of several *AMY* genes in leaves was more abundant in Koshihikari than in Takanari at many time points, but the α-amylase activity was not significantly different between the two varieties over the regrowth cycles. A similar trend was also observed in stubble. These data imply that α-amylase activity may not be regulated at the mRNA accumulation level. Cereal seeds, including rice, contain α-amylase inhibitors, likely to regulate seed development and germination [30,31]. A similar mechanism may be involved in regulating starch catabolism in vegetative tissues.

Cytokinin regulates growth, metabolism, nutrient absorption, and transport [26,27], all of which appear to be linked with regrowth vigor upon defoliation. The relationship between cytokinin and regrowth has been investigated in forage and turf grasses. In perennial ryegrass (*Lolium perenne*), defoliation decreased the concentrations of bioactive cytokinins such as trans-zeatin (tZ) and trans-zeatin riboside (tZR) but increased the content of another cytokinin form, cis-zeatin (cZ), in leaf sheaths [32]. In annual ryegrass (*L. multifolorum*), the first defoliation increased the concentrations of ZR in new leaves and stubble, but continuous defoliation decreased the ZR levels in both tissues [19]. Based on these results, it is still unclear whether defoliation increases or decreases bioactive cytokinin levels. The effect of cytokinin on vegetative regrowth is also ambiguous. Indeed, it was shown that the exogenous application of a synthetic cytokinin, 6-BA, increased vegetative regrowth in annual ryegrass relative to the non-spray controls [33]. Conversely, 6-BA spray reduces the biomass of newly emerged leaves in annual ryegrass, depending on the time of the spray [34]. These two studies also differed in the frequency of 6-BA spray and plant age. Collectively, these results demonstrate that the effect of defoliation on cytokinin accumulation and the impact of cytokinin application on vegetative regrowth depend on plant age, frequency of defoliation, and frequency and time of cytokinin application. 

The contradictory effect of cytokinin on shoot growth and physiology has been recognized in non-defoliated rice and other plants. Generally, cytokinin increases shoot cell growth, chlorophyll accumulation, and photosynthetic activity [26,27]. However, loss-of-function mutations of *DECUSSATE* in rice and *ABERRANT PHYLLOTAXY1* in maize, both of which are positive regulators for cytokinin signaling, increased the size of SAMs with enhanced cell division activity even though cytokinin signaling was inhibited [35,36,37]. It is anticipated that the role of cytokinin in shoot growth and physiology is determined by cell phases at which cytokinin accumulation is promoted. When cytokinins were induced in the cell expansion phase through upregulation of the agrobacterial isopentenyl transferase gene (*ipt*) and downregulation of the barley cytokinin oxidase/ dehydrogenase gene (*HvCKX2*) by a chemically inducible promoter, the leaf size, chlorophyll content, and photosynthetic activity increased in Arabidopsis [38]. In contrast, cytokinin excess in the cell division phase suppressed cell expansion, chlorophyll biogenesis, and photosynthesis. The present study demonstrates that cytokinin can serve as a negative regulator for vegetative regrowth upon defoliation (Figure 7). These data were obtained from plants exposed to cytokinin daily and mutants in which cytokinin biosynthesis is constitutively inhibited. In addition, these plants were subjected to periodic defoliation for 3–4 weeks. Under these conditions, cytokinin can first promote the growth of elongating leaves because most leaf cells are in the expansion phase. However, after the maturity of the elongating leaves, cytokinin suppresses the growth of the next and following leaves because the majority of leaf cells are in the cell division phase. Altogether, these data imply that properly regulating cytokinin levels in the cell division and expansion phases in shoots is necessary for enhanced vegetative regrowth upon defoliation.

## 4. Materials and Methods

### 4.1. Plant Materials and Growth Conditions

Rice (*Oryza sativa* L.) cv. Koshihikari, Takanari, and Nipponbare were used for this study. A *log-6* knockout mutant in the Nipponbare background, which does not synthesize active cytokinin, was also analyzed in this study [28]. Seeds were sterilized in 3% (*w*/*v*) sodium hypochlorite and 0.1% (*v*/*v*) Tween-20 for 10 min and rinsed thoroughly with water. Sterilized seeds were germinated on wet paper towels for 5 days at 28 °C under 12 h light (270 µmol/m^2^/s; fluorescent tubes, FL40S-W R F3, Panasonic, Osaka, Japan) and 12 h dark cycles in an incubator. Seedlings were transplanted into pots (W × L × H = 92 mm × 92 mm × 82 mm) containing a pre-fertilized soil mix (Kumiai rice seedling soil, No. 3, Takara Industry, Gunma, Japan) with 9 seedlings per pot. Plants were grown at 28 °C under 12 h light (400 µmol/m^2^/s; LED lamps, HS180W/BGR001, Espec Mic, Osaka, Japan) and 12 h dark cycles in a growth chamber. Pots were placed in containers filled with water, and water was supplied daily on the soil surface using a watering can. Light intensity was measured by a light meter, LI-250A, equipped with a quantum sensor, LI-190R (LI-COR Biosciences, NE, USA).

After 14 days, plants were cut to 2.5 or 5 cm above the soil surface with scissors at midday and allowed to regrow for 4 or 7 days in a growth chamber under the conditions described above. Weekly defoliation–regrowth cycles were repeated up to four times. In each cycle, defoliated leaves and stubble were harvested, immediately frozen in liquid nitrogen, and stored at −80 °C until use. For some experiments, defoliated leaves and stubbles in the third or fourth regrowth cycle could not be analyzed, because sufficient tissues were not obtained for biochemical and photosynthesis analyses.

### 4.2. Carbohydrate Assays

Total soluble carbohydrates were quantified using the method of [39]. Frozen tissues (50 mg) were homogenized in 1 mL of 80% (*v*/*v*) ethanol and incubated at 80 °C for 20 min. Following centrifugation, the supernatant was transferred to a new tube. The extraction process was repeated twice more, with the three extracts combined and dried under a vacuum. After rehydration in 1 mL of water, total soluble carbohydrates were determined by the anthrone method, with glucose as the standard [20]. The extract (25 µL) was mixed with 1 mL of 0.14% (*w*/*v*) anthrone solution in 100% sulfuric acid. Following the reaction at 100 °C for 20 min, A_620_ of the solution was determined with a spectrophotometer.

The starch content was assayed by the method of [40]. The pellet obtained after ethanol extraction was washed with water and resuspended in 1 mL of water containing 10 units of heat-resistant α-amylase. The suspension was incubated at 95 °C for 15 min. After cooling, 25 µL of 1 M sodium citrate (pH 4.8) and 5 units of amyloglucosidase were added to the suspension. Following incubation at 55 °C for 1 h, the reaction mixture was centrifuged for 30 min, and the supernatant (100 µL) was subjected to the anthrone assay as described above.

### 4.3. Quantitative RT-PCR Analysis

Total RNA was extracted from frozen tissues (100 mg) using an RNeasy Plant Mini kit (Qiagen, Hilden, Germany). Genomic DNA was eliminated by on-column digestion using DNase I (Qiagen). cDNA (20 µL) was synthesized from 1 µg of total RNA using a PrimeScript RT reagent kit (Takara Bio, Shiga, Japan). Oligo dT was used as a primer. Real-time PCR was performed in a 15 µL reaction using FastStart Essential DNA Green Master (Roche, Basel, Switzerland) in the LightCycler 96 System (Roche). Amplification specificity was validated by melt-curve analysis at the end of each PCR cycle. Relative transcript abundance was calculated using the comparative cycle threshold method. *ACTIN1* (Os03g0718100) and *UBIQUITIN1* (Os06g0681400) were used as normalization controls. Sequences and annealing temperatures of primer pairs are listed in Table S1.

### 4.4. Enzyme Assays

The sucrose synthase activity was assayed in the sucrose cleavage direction by measuring the UDP-dependent production of fructose with the enzymatic determination of fructose. Frozen tissue (50 mg) was homogenized in 0.4 mL of cold extraction buffer containing 100 mM HEPES-KOH (pH 7.5), 1 mM ethylenediaminetetraacetic acid (EDTA), 5 mM MgCl_2_, 5 mM dithiothreitol, 10 mM NaHSO_3_, and 1 mM phenazine methosulfate. Following centrifugation for 20 min at 4 °C, the supernatant was desalted with a PD MiniTrap G-25 desalting column (Cytiva, MA, USA) equilibrated with the extraction buffer (2 mL per sample) at 4 °C. A reaction mixture (450 µL) containing 50 mM HEPES-KOH (pH 7.0), 2 mM MgCl_2_, 1 mM EDTA, 15 mM KCl, 25 mM sucrose, and 1 mM UDP was mixed with 50 µL of the desalted extract and incubated at 30 °C for 30 min. For blank reactions, UDP was omitted from the reaction mixture. The reaction was stopped by heating at 100 °C for 2 min. Fructose was quantified enzymatically by coupling to the reduction of NADP^+^. The reaction mixture (400 µL) was combined with the assay buffer (600 µL) containing 50 mM HEPES-KOH (pH 7.0), 8 mM MgCl_2_, 1.5 mM ATP, 2 mM NADP^+^, 1 U/mL of hexokinase, 4 U/mL of phosphoglucose isomerase, and 1 U/mL of Glucose-6-phosphate dehydrogenase and incubated at 30 °C for 15 min. After the reaction, A_340_ was measured using a spectrophotometer. One unit of sucrose synthase is defined as the amount of enzyme required to release 1 µmol of fructose in 1 min. 

The activity of α-amylase was determined using an α-amylase assay kit (Kikkoman, Tokyo, Japan). Frozen tissue (50 mg) was homogenized in 400 µL of a cold extraction buffer containing 50 mM sodium acetate (pH 5.3) and 0.5 mM CaCl_2_. The extract was slowly agitated at 4 °C for 3 h. The homogenate was centrifuged twice at 4 °C for 15 min. The supernatant (90 µL) was added to a reaction mixture containing 150 µL of the substrate solution and 150 µL of the enzyme solution in the kit. The mixture was incubated at 37 °C for 20 min, and the reaction was stopped by adding 600 µL of the stop solution. A_400_ of the reaction was measured using a spectrophotometer. One unit of α-amylase is defined as the amount of enzyme required to release 1 µmol of 2-chloro-4-nitrophenol (CNP) in 1 min. 

### 4.5. Chlorophyll Content and Fluorescence Measurements

Chlorophyll content was quantified from 50 mg of defoliated leaves in 5 mL of 100% methanol, as described by [41]. Following centrifugation at 4 °C for 20 min, A_652.0_ and A_665.2_ of the supernatant were measured with a spectrophotometer.

Chlorophyll fluorescence was determined on the fully opened, uppermost leaves of regrown plants using a portable chlorophyll fluorometer (MINI-PAM-II; WALZ, Effeltrich, Germany) equipped with a leaf-chip holder. Following a 30 min dark adaptation using a dark leaf clip, leaves were exposed to continuous actinic light (1500 µmol/m^2^/s) for 5 min, and then chlorophyll fluorescence parameters were determined. The effective quantum yield of photosystem II photochemistry (Ø*_PSII_*) and photochemical quenching (*qP*) were calculated as (*F*′*_m_* − *F*′)/*F*′*_m_* and (*F*′*_m_* − *F*)/(*F*′*_m_* − *F*′_0_) [42]. 

### 4.6. Cytokinin Response Assays

For cytokinin treatment experiments, Koshihikari and Takanari at 14 days old were exposed to periodic defoliation as described in Section 4.1. Upon the first defoliation, a solution containing 100 or 200 µM of a synthetic cytokinin, 6-benzyl adenine (6-BA), in 0.1% (*v*/*v*) dimethyl sulfoxide (DMSO) or mock solution (0.1% (*v*/*v*) DMSO) was sprayed onto plants. Spray treatment was continued on a daily basis until the end of the fourth regrowth cycle. For cytokinin biosynthesis mutant analysis, a *log-6* knockout mutant [28] and wild-type (cv. Nipponbare) plants grown for 14 days were subjected to weekly defoliation as described in Section 4.1. For both cytokinin treatment and mutant analysis, shoot tissues regrown from defoliated plants were harvested, dried, and weighed.

### 4.7. Statistical Analyses

Statistical analyses were carried out using SPSS Statistics 23 (IBM, New York, NY, USA). Student’s *t*-test was performed to compare two datasets. For multiple mean comparisons, ANOVA with Tukey’s honest significant test was utilized. 

## 5. Conclusions

This study has confirmed and identified physiological and molecular traits associated with vegetative regrowth upon defoliation using rice as a model. Such traits include photosynthesis by newly emerged leaves, a reduced and increased transcript accumulation of SUS I and SUS III subfamilies, respectively, reduced sucrose synthase activity, and dampened sensitivity to cytokinin. Most of these traits have been recognized owing to the usage of rice as plant materials. The data presented here and in past publications suggest that vital components influencing regrowth vigor are the de novo production of carbohydrates by newly emerged leaves, the proper management of carbohydrate reserves in leaves and stubble, and the fine-tuning of cytokinin synthesis and signaling in shoots. Due to the conserved status of regulatory mechanisms underlying growth and development within the grass family (*Poaceae*) [15,16,17], the findings obtained in this study can be applicable to identify target genes and marker phenotypes used for the development of new varieties with enhanced regrowth vigor in forage and turf grasses.

## Data Availability

The data are contained within the manuscript and Supplementary Materials.

## Supplementary Materials

The following supporting information can be downloaded at: https://www.mdpi.com/article/10.3390/ijms25105070/s1. References [25,43,44,45] are cited in the supplementary materials.
