# Supplementary material for: Possible Roles of Carbohydrate Management and Cytokinin in the Process of Defoliation–Regrowth Cycles in Rice
Source: Int J Mol Sci. 2024 May 7;25(10):5070. doi: 10.3390/ijms25105070 (PMC11120658; doi:10.3390/ijms25105070)
Supplement: Supplementary file 1 [file ijms-25-05070-s001.zip › ijms-2957892-supplementary.pdf]

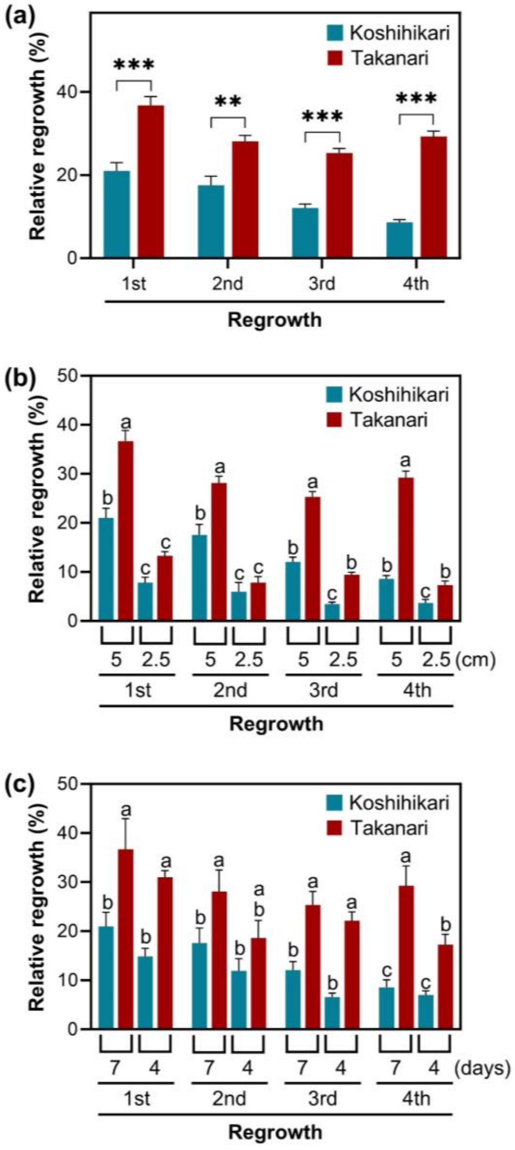

Figure S1. Regrowth vigor after defoliation based on leaf areas. (a) Relative regrowth of Koshihikari and Takanari after defoliation cycles. Fourteen-day-old plants were exposed to weekly clipping at 5 cm cutting height and regrown for 7 days. The defoliation-regrowth cycles were repeated four times. Relative regrowth was calculated by comparison with the total leaf area of clipped leaves at the first defoliation in each variety. The data represent means ± SE from four replicates. Asterisks indicate significant differences between the two varieties (t-test; $^{**}P < 0.01$, $^{***}P < 0.001$). (b) Relative regrowth of Koshihikari and Takanari after defoliation at 5 or 2.5 cm cutting height. Plants were exposed to defoliation cycles every 7 days at distinct cutting heights. (c) Relative regrowth of Koshihikari and Takanari after defoliation at a 7- or 4-day interval. Plants were exposed to defoliation cycles at 5 cm cutting height at distinct regrowth intervals. In (b) and (c), Data represent means ± SE from four replicates. Bars not sharing the same letter significantly differ in each defoliation cycle (ANOVA followed by Tukey HSD test; $P < 0.05$).

Table S1. Primer sequences and annealing temperature used for quantitative RT-PCR analysis.

	Gene name	Gene ID	Forward primer	Reverse primer	Annealing temperature	References
SUS1	*SUCROSE SYNTHASE 1*	Os03g0401300	CATCTCAGGCTGAGACTCTGA	CAAATTCAATCGACCTTACTT	52	[1]
SUS2	*SUCROSE SYNTHASE 2*	Os06g0194900	GAGGCTGATGACCTTGACTGG	CCCTCCATTACTTGGATGTGCT	59	[1]
SUS3	*SUCROSE SYNTHASE 3*	Os07g0616800	GTGAGACACGCCGCTACCTGGAG	CCCTGAAATCAACTAAACCCT	55	[1]
SUS4	*SUCROSE SYNTHASE 4*	Os03g0340500	TGAACTGGCGAAGACTGTACC	CAATGGCAATGAAGCACTGAG	57	[1]
SUS5/SUS7	*SUCROSE SYNTHASE 5/7*	Os04g0309600 / Os04g0249500	CAGGTTAGCCGGGCAGAAG	CATTGTACACAAATCTCACGCAAG	57	[1]
SUS6	*SUCROSE SYNTHASE 6*	Os02g0831500	CGAACAACCAGCACAACCTAC	GGAGCAGTGGTCCAAGTAAG	57	[1]
AMY1C	*α-AMYLASE 1C*	Os02g0765400	TATCATGGAGGCTGACAGCG	GCTAATTGTGCCTCTCCACC	58	[2]
AMY2A	*α-AMYLASE 2A*	Os06g0713800	GCCGATCATCGCACCTCTTC	CGATCCCACATATCAGTGACG	57	[2]
AMY3A	*α-AMYLASE 3A*	Os09g0457400	GAGGGTCATCACCAAGATCG	TGTGTAGCTAGCTTGCGAGC	57	[2]
AMY3C	*α-AMYLASE 3C*	Os09g0457800	TCCATGTCGTTGCTCACGGC	CGCCCTGCTGGAACTCTAAGAC	61	[3]
AMY3D	*α-AMYLASE 3D*	Os08g0473900	CGGGATAGTCATGCTCAAAC	ACGCTACAATCGGATACAAAA	55	[4]
AMY4A	*α-AMYLASE 4A*	Os01g0715400	TCGCCTGAAGGATACATGCC	GATTCAGGACGGCATCACCA	59	[2]
AMY5A	*α-AMYLASE 5A*	Os04g0403300	TCAGGGTCATTGGCCATTCC	TGGGGATGCCAGGATGAGTA	59	[2]
ACT1	*ACTIN 1*	Os03g0718100	CCGAGCGGGAAATTGTGAG	CTATGAAGGAAGGCTGGAAGAGG	58	[4]
UBQ1	*UBIQUITIN 1*	Os06g0681400	CAGTAAGTCCTCAGCCAT	AGACCAGACAACCATAGC	53	[1]

1. Fan, C.; Wang, G.; Wang, Y.; Zhang, R.; Wang, Y.; Feng, S.; Luo, K.; Peng, L. Sucrose synthase enhances hull size and grain weight by regulating cell division and starch accumulation in transgenic rice. *Int. J. Mol. Sci.* **2019**, *20*, 4971.
2. Liu, X.; Luo, J.; Li, T.; Yang, H.; Wang, P.; Su, L.; Zheng, Y.; Bao, C.; Zhou, C. *SDG711* is involved in rice seed development through regulation of starch metabolism gene expression in coordination with other histone modifications. *Rice* **2021**, *14*, 25.
3. Sugimura, Y.; Michiyama, H.; Hirano, T. Involvement of α-amylase genes in starch degradation in rice leaf sheaths at the post-heading stage. *Plant Prod. Sci.* **2015**, *18*, 277-283.
4. He, Y.; Zhu, M.; Li, Z.; Jiang, S.; He, Z.; Xu, S.; Chen, X.; Hu, Z.; Zhang, Z. *IPA1* negatively regulates early rice seedling development by interfering with starch metabolism via the GA and *WRKY* pathways. *Int. J. Mol. Sci.* **2021**, *22*, 6605.
